# Traumatic Epiglottitis following a Blind Insertion of the Hand during Convulsion

**DOI:** 10.1155/2018/8398502

**Published:** 2018-08-28

**Authors:** Koji Yokoyama

**Affiliations:** Department of Pediatrics, Japanese Red Cross Wakayama Medical Center, Wakayama, Japan

## Abstract

Traditionally, it has been recommended that first-responders should place chopsticks or their hand in a child's mouth to prevent the child from biting their tongue during convulsion. The practice persists locally in parts of Japan and can cause adverse events. We report a traumatic epiglottitis following the thrusting of a guardians' hand into a 13-month-old girl's mouth to prevent her from biting her tongue.

## 1. Case Presentation

Inappropriate maneuvers persist in cardiopulmonary resuscitation. For example, the prevention of and relief from “tongue swallowing” appears to take priority over chest compression [1]. In Japan, it is recommended traditionally that the first-responder should place chopsticks or their hand in a child's mouth to prevent biting of the tongue during convulsions. This practice is used locally in parts of Japan, and can cause adverse events. Here, we report a case of traumatic epiglottitis following the thrusting of a guardian's hand into the mouth of a small girl to prevent her from biting her tongue.

**Figure 1:**
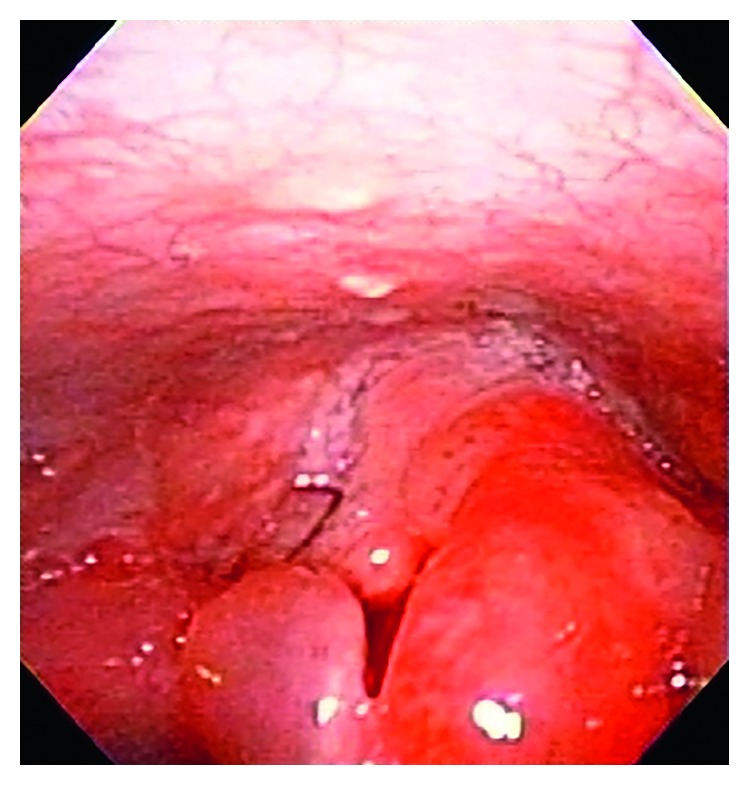
Image from the laryngoscopic examination showing the left side erythema and edematous epiglottis and aryepiglottic folds.

A 13-month-old female was admitted to our emergency department because of sursumvergence and disturbance of consciousness. She received hypothermia therapy following an incidence of neonatal asphyxia during the newborn period. She did not have a history of convulsive seizures. She was appropriately vaccinated for her age, including against *Haemophilus influenza* type B. The child's grandmother found her unconscious and attempted to prevent the child from biting her tongue by forcefully opening her mouth and placing her fingers in the child's mouth. At presentation at the emergency room, the patient was conscious and alert, afebrile, and showed vesicular breath sounds and no rale. Her temperature was 36.9°C, respiratory rate was 45 breaths/min, pulse rate was 142 beats/min, and blood pressure was 130/80 mmHg. After a few hours, the patient developed irritability, drooling, inspiratory stridor, and sternal retraction. Enhanced computed tomography showed no foreign bodies in the airway or esophagus. She appeared ill, irritable, and in moderate respiratory distress, which did not improve with racemic epinephrine and systemic corticosteroid therapy. The otolaryngologist service performed a laryngoscopic examination, which revealed left side erythema and an edematous epiglottis and aryepiglottic folds consistent with acute epiglottitis (Figure 1). The patient was admitted to the intensive care unit. An intubation tube was placed without difficulty, and she was treated with intravenous antibiotics and corticosteroid therapy. On day four after intubation, the patient's epiglottis appeared normal by laryngoscopic examination, and she was subsequently extubated. Following overnight observation, the child was discharged to home at 6 days after her emergency intubation. Cultures of the epiglottis and blood were negative.

## 2. Discussion

Despite a reduced incidence of infectious epiglottitis following the introduction of the *Haemophilus influenza* type B conjugate vaccine, physicians must pay attention to this life-threatening cause of airway obstruction [2–4]. Previously, there were reports of traumatic epiglottitis following forceful insertion of a guardian's hand into the mouth to remove foreign bodies including fatal outcomes [5–8]. Our case illustrates physicians should be aware of the possibility of inappropriate cardiopulmonary resuscitation manipulation related acute epiglottis. In addition, there is urgent need to ensure the use of appropriate cardiopulmonary resuscitation maneuvers, especially in young children.
